# The Role of Perceptual Interference, Semantic Interference, and Relational Integration in the Development of Analogical Reasoning

**DOI:** 10.3389/fpsyg.2020.00756

**Published:** 2020-05-12

**Authors:** Xiao Yu, Liuna Geng, Yinghe Chen, Congcong Han, Xiaojing Zhu

**Affiliations:** ^1^School of Developmental Psychology, Faculty of Psychology, Beijing Normal University, Beijing, China; ^2^School of Social and Behavioral Sciences, Nanjing University, Nanjing, China; ^3^School of Psychology, Shandong Normal University, Jinan, China; ^4^Department of Clinical Psychology, Yantai Affiliated Hospital of Binzhou Medical University, Yantai, China

**Keywords:** analogical reasoning, children, perceptual interference, relational integration, semantic interference

## Abstract

This study aimed to examine the role of perceptual interference, semantic interference, and relational integration (RI) in the development of analogical reasoning, and to compare the interactive pattern of interference and RI in children and adults. In Experiment 1, we tested 31 3- and 4-year-olds, 27 5- and 6-year-olds, and 40 adults for perceptual interference and RI in analogical reasoning. Perceptual interference emerged when proper mapping between analogically matching objects was incoherent with their perceptual features. RI was evaluated via manipulation of the number of objects in an analogical scene. Significant main effects of perceptual interference and RI were found in children and adults. In Experiment 2, we tested 30 3- and 4-year-olds, 27 5- and 6-year-olds, and 40 adults for semantic interference and RI in analogical reasoning. Semantic interference emerged when proper mapping between analogically matching objects was incoherent with their categorical features. Results showed significant main effects of semantic interference and RI in children and adults. The results of both experiments suggested different mechanisms of interference and RI in children and adults. For children, interference and RI depended on shared cognitive sources. If one factor (i.e., interference resolution) needed more cognitive demand, there would be limited resources available for another factor (i.e., RI). Furthermore, for adults, the increased load of RI and interference on adults’ analogical reasoning exceeded the sum of their respective singular effects. For 3- and 4-year-olds, the degree of perceptual interference was larger than the degree of semantic interference in the Binary Relation condition, whereas there was no significant difference between the degree of two types of interference in the Quaternary Relation condition. Moreover, for 5- and 6-year-olds, the degree of semantic interference was larger than the degree of perceptual interference in both relation conditions. For adults, there was no difference between the degree of two types of interference in both relation conditions. The article also discusses the theoretical and practical implications of this research.

## Introduction

Analogical reasoning is an important component of higher cognitive development in children. It refers to a conceptual strategy in which a source object is represented as similar to a target object, and correspondences are mapped between the two analogs (Gentner, 1983; Vendetti et al., 2017). There is general agreement that analogical reasoning is a fundamental skill that develops dramatically during childhood. It enables children to transfer learning across contexts and to understand novel situations (Rattermann and Gentner, 1998; Richland and Burchinal, 2013; Richland et al., 2016). Previous studies have focused on the effects of different cognitive capacities on analogical reasoning. These cognitive capacities include interference resolution (i.e., the ability to suppress irrelevant or conflicting information), relational integration (RI) (i.e., the ability to maintain and process information or rules related to the current task in working memory), and so on (Richland et al., 2006; Thibaut and French, 2016; Simms et al., 2018; Starr et al., 2018).

However, the aforementioned studies manipulated only one type of interference (e.g., perceptual interference or semantic interference) in a single group (e.g., children or adults). The interactive effects of different types of interference and RI may differ for children and adults. To address this possibility, the current study examined the simultaneous contributions of two types of interference and RI on the development of analogical reasoning. It also compared the different interactive patterns of interference and RI in children and adults.

### Perceptual and Semantic Interference in Analogical Reasoning

In well-controlled experiments, researchers usually manipulated interference to measure inhibitory control. Such studies have found that participants are affected by such manipulation. Contemporary studies have examined two types of interference: perceptual interference and semantic interference. Studying perceptual interference usually involves placing competing and superficially similar objects in different relational roles in the source and target scenes.

Many studies have used the Scene Analogy Task, in which children have to find the same pattern in two pictures (Richland et al., 2006; Whitaker et al., 2017; Simms et al., 2018). In Richland and colleagues’ study, perceptual interference was incorporated into a scene analogy task. A base scene showed a cat chasing a mouse, whereas the target scene showed a boy chasing a girl and also included a perceptually similar item (e.g., a sitting cat) or a dissimilar item (e.g., a sandbox). The experimenters asked children to identify an object in the target scene that was similar to the chased object in the base scene. They found that the children made more errors when the interference was perceptually similar to the chased object than when the interference was perceptually dissimilar.

Another type of interference is semantic interference. Semantic interference involves incoherence on an abstract concept level (Holyoak and Thagard, 2010). Such incoherence occurs when proper mapping violates the consistency of a reasoner’s belief about the mapped objects or when these objects do not fulfill their stereotypical functions (Green and Hummel, 2003). Some researchers have tended to operationalize semantic interference as cross-mapping between object categories and the relational roles to which they are bound.

Chuderska and Chuderski (2014) used a Scene Analogy Task to investigate the role of semantic interference in analogical reasoning in adults. They manipulated semantic interference by placing objects in corresponding scenes in different relational roles. The source analogical scene was a smashed glass bottle damaging an inflatable blown ball, whereas the target scene was a heavy book damaging a mirror. Thus, the bottle and the mirror are both made of glass, but fulfill opposing roles. The researchers found that semantic interference with the key structure in the pictorial analogical scene decreased adults’ analogical mapping accuracy. Other developmental studies have also demonstrated the effect of semantic interference on analogical reasoning. Thibaut et al. (2010b) assessed the performance of 3- to 4-year-old children on semantic A:B:C:? problems, varying the number of cases of interference (either one or three) and the association strength (strong or weak association between A and B, and C and the distractor). They found that children’s responses dropped sharply in trials with three cases of semantic interference, especially when the association strength was weaker.

However, there are several problems with the aforementioned studies. First, the Scene Analogy Task (Richland et al., 2006; Whitaker et al., 2017; Simms et al., 2018) used the same object (e.g., the cat in Richland et al., 2006) in a different role in the source and target scenes, as a type of cross-mapping manipulation. However, this confounded perceptual interference and semantic interference, meaning that it is not possible to infer that the children’s errors are totally due to perceptual interference. Second, the manipulation of perceptual interference usually involved varying superficial similarities (such as the color and shape of objects) that can be identified by infants (Siegel and Linda, 1979; Adler, 1996). However, semantic interference is more strongly associated with the conceptual level of declarative knowledge, which is more strongly influenced by educational experience (Mark et al., 1978). It would be informative to answer the question of how do children go from being affected by superficial perceptual interference to being affected by the more abstract semantic interference. In fact though, very few researchers have attempted to manipulate both types of interference at once. To our knowledge, no study has focused on perceptual interference and semantic interference in children’s analogical reasoning. Thus, the current study is the first to introduce both types of interference in order to compare the simultaneous effects of different types of interference on the development of analogical reasoning.

### Interference With Relational Integration in Analogical Reasoning

Besides resolving interference, analogical reasoning also depends on integrating multiple relations (Viskontas et al., 2004, 2005). In order to make a correct analogy, individuals must consider simultaneous relations in their minds. More generally, researchers have defined RI as the number of relations or objects that a reasoner must simultaneously “hold in mind” to integrate in an analogical process (Halford et al., 2005). For example, in the previously mentioned Scene Analogy Task (Richland et al., 2006), the researchers also manipulated the relation in the scene. In the one-relation condition, children saw a cat chasing a mouse and a boy chasing a girl. In the two-relation condition, children saw a dog chasing a cat and the cat chasing a mouse; and a mother chasing a boy and the boy chasing a girl. In the one-level relation condition, children need to consider only one “chasing” relation in order to solve the task correctly. In the complicated two-level relation condition, however, the children must integrate two “chasing” relations simultaneously. Children, particularly those in the younger age groups, performed more poorly in the two-relation condition than in the one-relation condition.

A range of empirical studies have evaluated the role of RI in analogical reasoning. For instance, one study using the People Pieces Analogy task (PPA task) varied the number of goal-relevant traits as the measure of RI (Cho et al., 2007). The analogical reasoning problem included two cartoon characters that were described according to four binary dimensions: clothing color (black or white), gender (male or female), height (tall or short), and width (wide or narrow). Participants were asked to compare the relationships of these dimensions between two pairs of characters. They were asked to match one to four of the dimensions and ignore the other dimensions. If any of the to-be-matched-to relations were different across the pairs, participants were to respond with “different,” and if all of the to-be-matched-to relations were the same, they were to respond with “same.” This study found a main effect of RI in adults. Similar results were observed in a study by Chuderska and Chuderski (2014). This study involved the manipulation of the number of objects in an analogical scene to evaluate RI. In the Binary Relation condition, an inflatable ball damaged by a smashed glass bottle was used as the source scene, and a dollhouse damaged by a heavy book was used as the target scene. The Quaternary Relation condition extended the Binary Relation condition by using two additional objects to increase the RI. It included a table with a broken leg, which caused the glass bottle to fall and damage the ball: this was the source scene. In the target scene, a broken bookshelf allowed the heavy book to fall and damage the dollhouse. The results revealed that this RI manipulation significantly affected analogical reasoning performance.

How, then, can interference play a role in analogical reasoning with more complicated RI? Many previous studies have shown that children, adults, and the elderly all have difficulty in inhibiting irrelevant interference, especially when required to integrate multiple relations (Viskontas et al., 2004, 2005; Chuderska and Chuderski, 2014; Simms et al., 2018). However, there is no consensus in the literature as to the interactive mechanisms responsible. For example, Krawczyk et al. (2008) used a picture analogy task with a multiple-choice answer format to study patients with frontal-variant frontotemporal lobar degeneration. The results revealed no interaction between RI and interference. However, another computational account study (Morrison et al., 2011) suggested that the interactive effect of RI and interference exceeds the sum of their respective singular effects. This means that the ability to properly process increased RI and interference simultaneously leads to rapid exhaustion and the collapse of processing.

However, some studies of children’s perceptual interference and adults’ semantic interference (Richland et al., 2006; Chuderska and Chuderski, 2014) have demonstrated that the interactive effect of RI and interference is less than the sum of their respective singular effects. This means that analogical reasoning performance is only disrupted by interference when RI does not exceed working memory capacity. Thus, the different interactive effects have hinted at a different mechanism underlying interference and RI in analogical reasoning.

The differences in results in recent studies may have been due to different experimental paradigms, different age groups being studied, and different types of interference. Thus, the current study aimed to evaluate and compare the mechanisms of the interaction of two types of interference and RI in children and adults.

### The Current Study

The current study adopted the Scene Analogy Task (Chuderska and Chuderski, 2014). In contrast with studies evaluating a single type of interference (for example, only perceptual interference is investigated in Richland’s Scene Analogy Task [2006] or Cho et al.’s PPA task [2007] and only a more abstract conceptual semantic interference is investigated in Chuderska and Chuderski’s SAT task [2014]), we introduced both perceptual interference and semantic interference to compare the effects of different types of interference on the development of analogical reasoning. Furthermore, in contrast with similar studies that evaluated specific age groups, such as children (Richland et al., 2006), adults (Chuderska and Chuderski, 2014), or patients (Krawczyk et al., 2008), our study evaluated both children and adults. The aim of including children and adults was to enhance the generalization of our results. Moreover, previous studies have shown that interference, especially perceptual interference, in the analogical scene can significantly decrease the performance of preschool-age children, while this effect can be eliminated or even disappear in school-age children or adolescents (Richland et al., 2006; Simms et al., 2018). Thus, the current study included preschool children as participants.

In the current study, we tested the effects of perceptual interference and RI on analogical reasoning in Experiment 1. We manipulated four conditions in the Scene Analogy Tasks: Binary Relation with No Perceptual Interference, Binary Relation with Perceptual Interference, Quaternary Relation with No Perceptual Interference, and Quaternary Relation with Perceptual Interference. We assumed that there were main effects of perceptual interference and RI in the development of analogical reasoning. The interactive pattern of perceptual interference and RI was different for children and adults. In Experiment 2, we tested the effects of semantic interference and RI on analogical reasoning. We also manipulated four conditions in the Scene Analogy Task: Binary Relation with No Semantic Interference, Binary Relation with Semantic Interference, Quaternary Relation with No Semantic Interference, and Quaternary Relation with Semantic Interference. We assumed that there were main effects of semantic interference and RI on the development of analogical reasoning. The interactive pattern of semantic interference and RI was different for children and adults.

## Experiment 1

### Method

#### Participants

Thirty-one 3- and 4-year-old children (14 females; *M* = 43 months, *SD* = 5.33 months), 27 5- and 6-year-old children (14 females; *M* = 68.5 months, *SD* = 3.48 months), and 40 adults (22 females; *M* = 24.5 years, *SD* = 2.43 years) took part in this experiment. All participants were recruited in Beijing, China. They were all right-handed and reported normal or corrected-to-normal vision without color blindness. None of the subjects had previously participated in similar experiments. Informed written consent from participants or parents was obtained. Children were given gifts and adults were paid for their participation.

#### Materials

We adopted the Scene Analogy Tasks from Chuderska and Chuderski (2014). In the current analogical scene task, there were 27 key relation groups including four conditions with 27 paired scenes in each condition. All scenes depicted everyday instances of common relations (such as damaging, chasing, pulling, etc.). All items were similarly detailed. No key relations or objects were repeated between paired scenes. To ensure that these relations and objects were familiar to preschool children, we asked three kindergarten teachers to evaluate the scene items in Experiments 1 and 2. The Kendall coefficients of concordance of all the paired scenes were above 0.8. Furthermore, we asked 20 6-year-old children to describe all of the scenes in words. Experimenters evaluated these descriptions according to the following criterion: in each picture of paired of scenes, two points were given for correct descriptions of both the key analogical relations and the key objects. One point was given for a correct description of key analogical relations or the key objects. Zero point was given for incorrect descriptions of both the key analogical relations and the key objects. The mean evaluation score for each picture was 1.7, which suggested that children’s descriptions were essentially consistent with the pictures.

**FIGURE 1:**
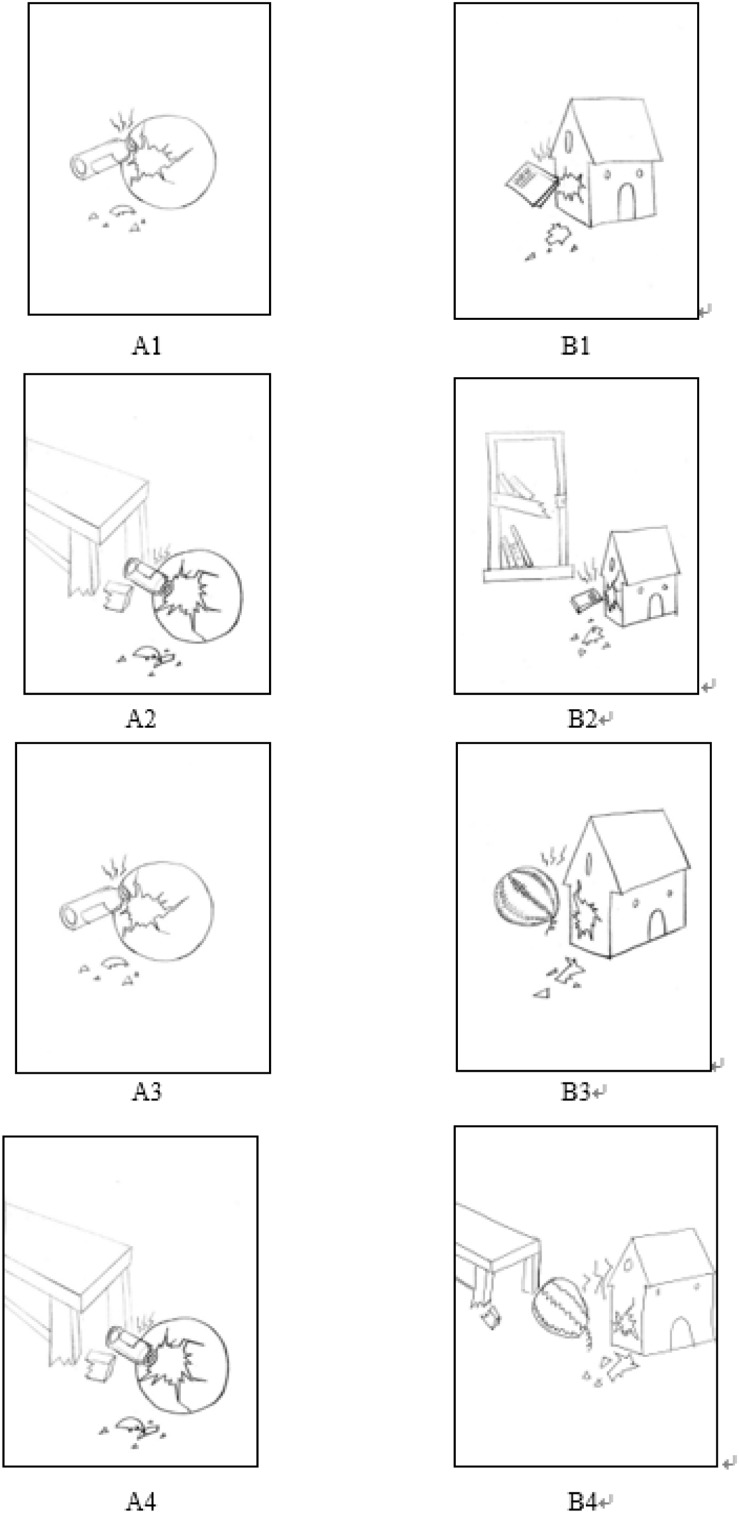
Examples of trials for the analogy scenes in Experiment 1. Pictures labeled with “A” represent source pictures and pictures labeled with “B” represent target pictures. **(A1,B1)** shows a Binary Relation with No Perceptual Interference analogy scene [**(A1)** is the source scene: a glass bottle damaged the ball; **(B1)** is the target scene: a heavy book damaged the dollhouse]; **(A2,B2)** shows a Quaternary Relation with No Perceptual Interference analogy scene [**(A2)** is the source scene: the table leg was broken, which caused the glass bottle to fall and damage the ball; **(B2)** is the target scene: the bookshelf was broken, which caused the heavy book to fall and damage the dollhouse]; **(A3,B3)** shows a Binary Relation with Perceptual Interference analogy scene [**(A3)** is the source scene: the same as **(A1)**; **(B3)** the target scene: a heavy watermelon damaged the dollhouse]; **(A4,B4)** shows a Quaternary Relation with Perceptual Interference analogy scene [**(A4)** is the source scene: the same as **(A2)**; **(B4)** is the target scene: the table leg was broken, which caused the heavy watermelon to fall and damage the dollhouse].

Figure 1 depicts an example of four conditions of one key relation group in Experiment 1 (Binary Relation with No Perceptual Interference, Binary Relation with Perceptual Interference, Quaternary Relation with No Perceptual Interference, and Quaternary Relation with Perceptual Interference). In half of the trials, each scene included a binary relation, whereas the remaining ones contained a quaternary relation (RI factor). The Binary Relation condition showed the relation between two items, such as a smashed glass bottle damaging an inflatable ball or a heavy book damaging a dollhouse. Quaternary relations were created from the binary relations by adding two more objects, such as a table with a broken leg, which caused a glass bottle to fall and damage a ball; or the bracket of a shelf being broken, which caused a book to fall and damage a dollhouse. In half of the trials, one object from a relation in the source scene was perceptually similar to another object included in that relation in the target scene (the perceptual interference factor). For example, the damaged ball in the source picture was perceptually similar to the watermelon causing the damage in the target picture. In the other half of the trials, there was no perceptual similarity in objects between the source scene and the target scene.

Perceptual interference emerged when proper mapping between analogically matching objects was incoherent with their perceptual features. The perceptual interference objects were inanimate objects (such as a watermelon) or animate objects (such as animals). The spatial locations of the extra objects and perceptual interference objects were controlled across paired of scenes. The relative sizes of the objects were also controlled across paired scenes. The relative sizes of the objects were varied to ensure that participants would not learn an analogical pattern. For example, the correct answer was not always an animate object or not always in the same location or not always a bigger object.

To ensure that the highlighted objects in source scenes were indeed perceptually similar to perceptual interference objects in target scenes, 12 undergraduates were asked to rate the perceptual similarity between the highlighted objects and other objects in the scene analogical tasks. For example, in the task in Figure 1, the instructions were as follows: Welcome to the picture evaluation! Please first look at all the pictures carefully. Then, please separately evaluate the perceptual similarity (such as similar shape, similar color, etc.) between the ball and the following objects: the glass bottle, the watermelon, the book, the bookshelf, the dollhouse, the table on a Likert seven-point scale. In all paired scenes, the highlighted objects were assessed as being perceptually similar to the perceptual interference objects with a score of 5.2 or more on the 1–7 scale. The highlighted objects were assessed as being perceptual similar to other objects (except for the perceptual interference objects) with a score of 2.7 or less on the scales. Furthermore, in order to clarify whether the highlighted objects were semantically similar to the perceptual interference objects or other objects, the 12 undergraduates were also asked to rate the semantic similarity between the highlighted objects and other objects in the scene analogical tasks. For example, in the take in Figure 1, the instructions were as follows: Please separately evaluate the semantic similarity (such as shared categorical features, etc.) between the ball and the following objects – the glass bottle, the watermelon, the dollhouse, the table, the book, the bookshelf on a Likert seven-point scale. The highlighted objects were assessed as being semantically similar to all objects (including perceptual interference objects) with a score of 2.4 or less on the scale. In accordance with the work of Thibaut et al. (2010b, 2011), these results indicated that the manipulation of perceptual interference was valid.

#### Procedure

Children were tested on paper with a size of 630 × 470 pixels. In Experiment 1, all children were asked to complete the practice stage first and then to perform the formal task. Instructions in the practice stage were as follows:

“We are going to play picture games. Let me show you how it works. On every page there are two pictures like this. There is a certain pattern in the left-hand source picture, and the same pattern occurs in the right-hand target picture. But it looks different. Let me show you what I mean. See the left-hand source picture: there is a smashed glass bottle that damaged an inflatable ball. And the right-hand target picture shows that a heavy book damaged a dollhouse (the experimenter pointed to each object as it was described.). Now, in this game, you first have to figure out what is the pattern that occurs in both pictures. Then I’m going to point to one thing in the left-hand source picture, and you tell me what is in the same part of the pattern in the right-hand target picture. So, on the first page, there is a smashed glass bottle and an inflatable ball in the left-hand picture. And there is a heavy book and a doll house in the right-hand picture. If I point to the ball, which one is in the same part of the pattern in the right-hand target picture?”

If children responded correctly, the experimenter gave feedback and then moved to the next analogy problem. If the children responded incorrectly, the experimenter gave feedback and then repeated the description of the relational objects in the source and target pictures. The experimenter then repeated the question. If the children again gave an incorrect answer, the experimenter gave the correct answer and moved to the next analogy problem. Children were supposed to complete two key relation groups including four conditions with two paired scenes in each condition in the practice stage. Specifically, each participant was supposed to complete one condition of each of the two groups, then a different condition of each of the two groups, until all four conditions were completed. The four conditions were presented in a counterbalanced order across participants in each age group. The two groups of each condition were presented in a random order. According to Richland et al. (2006), if a child fails in both groups of scenes, this indicates that the participants did not understand the relational instructions. Two children, 3 and 4 years old, were excluded for this reason.

The formal experiment stage was similar to the practice stage, but no detailed instructions or no feedback was given. In the formal experiment stage, another experimenter recorded the children’s responses. Children were supposed to complete 25 key relation groups including four conditions with 25 paired scenes in each condition. Specifically, each participant was supposed to complete one condition of each of the 25 groups, then a different condition of each of the 25 groups, until all four conditions were completed. The four conditions were presented in a counterbalanced order across participants in each age group. The 25 groups of each condition were presented in a random order. According to Richland et al. (2006), if a child refuses to provide an answer to more than five paired scenes, the child’s data should be excluded. In the current study, no children left more than five paired scenes blank. The total task lasted approximately 60 min for children. It was divided into four parts with proper breaks provided.

Adults were tested on a computer using E-prime 2.0. The adult experiments also included a practice stage with eight paired scenes and a formal experiment stage with 100 paired scenes. In the practice stage, participants were allowed to observe and explore both scenes for 40 s. Observation and exploration consisted of the subjects identifying objects, naming objects, and determining the pattern in the paired scenes. Then, the experimenter provided details regarding the pattern relation among the objects in the paired scenes. Second, one object was highlighted with a red arrow in the left-hand source picture for 30 s. Third, participants were asked to point to the corresponding object in the right-hand target picture using the mouse cursor. Then, feedback was given to the participants. In the formal experiment stage, there were also observation and exploration, highlighting, and response stages. No details or feedback was given in the formal experimental stage. The order of stimuli in the practice stage and the formal experiment stage was the same as in the children’s task. The total tasks lasted for approximately 2 h and was divided into four parts with proper breaks provided.

### Results

The descriptive statistics for participants’ accuracies are presented in Table 1. A 3 (Age Group: 3- and 4-year-olds, 5- and 6-year-olds, or adults) × 2 (RI: Binary Relations or Quaternary Relations) × 2 (Perceptual Interference: No Perceptual Interference or Perceptual Interference) mixed analysis of variance (ANOVA) was conducted on the accuracies. The results showed that all main effects were significant. The main effect of age group was significant, *F*(2,94) = 221.872, *p* < 0.001, η*^2^* = 0.825; 3- and 4-year-olds (*M* = 0.515 ± 0.011) responded correctly less frequently than 5- and 6-year-olds (*M* = 0.646 ± 0.011), and 5- and 6-year-olds responded correctly less frequently than adults (*M* = 0.805 ± 0.009). The main effect of RI was significant, *F*(1,94) = 171.399, *p* < 0.001, η*^2^* = 0.646; accuracy decreased when RI increased from two (*M* = 0.699 ± 0.007) to four (*M* = 0.612 ± 0.006) dimensions. The main effect of perceptual interference was also significant, *F*(1,94) = 420.041, *p* < 0.001, η*^2^* = 0.817; participants responded correctly less frequently when perceptual interference was present (*M* = 0.598 ± 0.007) than when it was absent (*M* = 0.712 ± 0.006).

**TABLE 1:** Mean accuracies and standard deviations of the Scene Analogy Task for 3- and 4-year-olds, 5- and 6-year-olds, and adults in Experiment 1.

Age	Binary relations		Quaternary relations	
		
	No perceptual interference	Perceptual interference	No perceptual interference	Perceptual interference
3- and 4-year-olds (*n* = 30)	0.676 ± 0.092	0.459 ± 0.113	0.507 ± 0.076	0.417 ± 0.091
5- and 6-year-olds (*n* = 27)	0.754 ± 0.069	0.616 ± 0.088	0.633 ± 0.074	0.582 ± 0.069
Adults (*n* = 40)	0.882 ± 0.076	0.806 ± 0.055	0.823 ± 0.059	0.710 ± 0.058

The interaction between age group and perceptual interference was significant, *F*(2,94) = 12.362, *p* < 0.001, η*^2^* = 0.208. A simple effect test in each perceptual interference condition (using a Bonferroni adjustment) showed a significant main effect of age group in the no perceptual interference condition, *F*(2,94) = 160.700, *p* < 0.001, η*^2^* = 0.774, as well as in the perceptual interference condition, *F*(2,94) = 204.516, *p* < 0.001, η*^2^* = 0.813. Tukey’s HSD tests showed that in both conditions, 3- and 4-year-olds performed worse than 5- and 6-year-olds and adults (*p*’s < 0.001); 5- and 6-year-olds performed worse than adults (*p*’s < 0.001). The interaction between age group and RI was not significant, *F*(2,94) = 1.916, *p* > 0.05.

**FIGURE 2:**
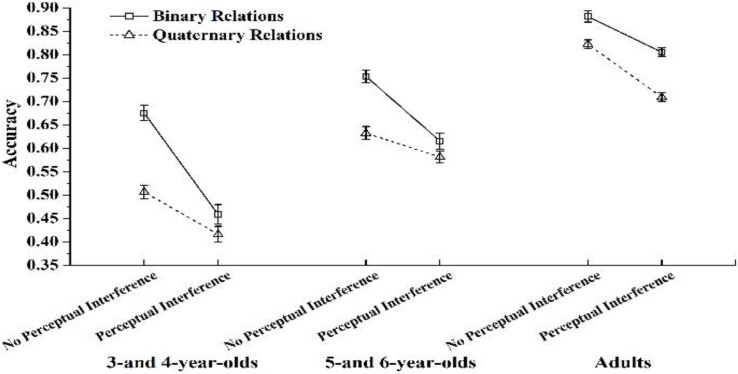
Accuracies as a function of relational integration and perceptual interference across age groups.

We also found a significant three-way interaction among age group, RI, and perceptual interference, *F*(2,94) = 19.563, *p* < 0.001, η*^2^* = 0.294. To further precisely characterize age-related differences in accuracy patterns on the task, a test of RI × perceptual interference was conducted on each age group separately (Figure 2).

Results showed that for 3- and 4-year-olds, there was a main effect of RI, *F*(1,29) = 70.589, *p* < 0.001, η*^2^* = 0.709; a main effect of perceptual interference, *F*(1,29) = 142.574, *p* < 0.001, η*^2^* = 0.831; and a significant interaction between RI and perceptual interference, *F*(1,29) = 42.262, *p* < 0.001, η*^2^* = 0.593. A simple effect test in each perceptual interference condition (using a Bonferroni adjustment) showed a significant main effect of RI in the no perceptual interference condition, *F*(1,29) = 147.972, *p* < 0.001, η*^2^* = 0.836, and in the perceptual interference condition, *F*(1,29) = 5.434, *p* < 0.001, η*^2^* = 0.158. Tukey’s HSD tests showed that in both perceptual interference conditions, children performed significantly worse when RI increased from two to four dimensions (*p* < 0.001 for the no perceptual interference condition; *p* < 0.05 for the perceptual interference condition).

For 5- and 6-year-olds, there was a main effect of RI, *F*(1,26) = 29.436, *p* < 0.001, η*^2^* = 0.531; a main effect of perceptual interference, *F*(1,26) = 162.414, *p* < 0.001, η*^2^* = 0.862; and a significant interaction between RI and perceptual interference, *F*(1,26) = 9.414, *p* = 0.005, η*^2^* = 0.266. A simple effect test in each perceptual interference condition (using a Bonferroni adjustment) showed a significant main effect of RI in the no perceptual interference condition, *F*(1,26) = 57.319, *p* < 0.001, η*^2^* = 0.688; and in the perceptual interference condition, *F*(1,26) = 2.076, *p* > 0.05. Tukey’s HSD tests showed that in the no perceptual interference condition, children performed significantly worse when RI increased from two to four dimensions (*p* < 0.001). In the perceptual interference condition, there was no significant difference between the Binary Relations condition and the Quaternary Relation condition (*p* > 0.05).

For adults, there was a main effect of RI, *F*(1,39) = 85.529, *p* < 0.001, η*^2^* = 0.687; a main effect of perceptual interference, *F*(1,39) = 150.461, *p* < 0.001, η*^2^* = 0.794; and a significant interaction between RI and perceptual interference, *F*(1,39) = 6.115, *p* < 0.05, η*^2^* = 0.136. A simple effect test in each perceptual interference condition (using a Bonferroni adjustment) showed a significant main effect of RI in the no perceptual interference condition, *F*(1,39) = 17.318, *p* < 0.001, η*^2^* = 0.308, and in the perceptual interference condition, *F*(1,39) = 179.353, *p* < 0.001, η*^2^* = 0.821. Tukey’s HSD tests showed that in both interference conditions, adults performed significantly worse when RI increased from two to four dimensions (*p*’s < 0.001).

We also calculated the difference in accuracy between no perceptual interference and perceptual interference in the Binary Relation condition and the difference in accuracy between no perceptual interference and perceptual interference in the Quaternary Relation condition across the three age groups. Paired *t* tests on these two differences in accuracy were conducted across the three age groups. The results showed that for 3- and 4-year-olds and 5- and 6-year-olds, the difference in accuracy scores between no perceptual interference and perceptual interference in the Binary Relation condition was significantly larger than that in the Quaternary Relation condition [for 3- and 4-year-olds, *t* (29) = 6.051, *p* < 0.001; for 5- and 6-year-olds, *t* (26) = 3.068, *p* = 0.005]. For adults, the difference in accuracy scores between no perceptual interference and perceptual interference in the Binary Relation condition was significantly smaller than that in the Quaternary Relation condition, *t* (39) = −2.473, *p* = 0.018.

These three patterns revealed changes with age. For 3- and 4-year-olds and 5- and 6-year-olds, the difference in accuracy between the perceptual interference and no perceptual interference conditions was larger when RI was simple (i.e., binary relations) rather than complex (i.e., quaternary relations). Similarly, the difference in accuracy between the binary relations and quaternary relations conditions was larger when perceptual interference was absent than when it was present. However, for adults, the corresponding difference was larger when RI was complex (i.e., quaternary relations) rather than simple (i.e., binary relations). Similarly, the difference in accuracy between the binary relations and the quaternary relations conditions was larger when perceptual interference was present than when it was absent.

The chance level differed across conditions (Richland et al., 2006). In the current study, the chance level ranged from 50% (Binary Relation with No Perceptual Interference and Binary Relation with Perceptual Interference) to 33% (Quaternary Relation with No Perceptual Interference and Quaternary Relation with Perceptual Interference). A paired *t* test demonstrated that 3- and 4-year-olds were above the level of chance for Binary Relation with No Perceptual Interference, *t* (29) = 10.434, *p* < 0.001; Quaternary Relation with No Perceptual Interference, *t* (29) = 12.517, *p* < 0.001; and Quaternary Relation with Perceptual Interference conditions, *t* (29) = 5.055, *p* < 0.001. Furthermore, the results of the 3- and 4-year-olds were not above the level of chance for the Binary Relation with Perceptual Interference, *t* (29) = −2.003, *p* = 0.055. This revealed that 3- and 4-year-olds understand the analogical relationships and can reason according to relational similarity to some extent. Moreover, the results of the 5- and 6-year-olds and adults were above the level of chance for all conditions.

### Discussion

Experiment 1 investigated the role of perceptual interference and RI in the development of analogical reasoning. The results revealed that manipulations of perceptual interference and RI resulted in significantly different results, which have several implications. First, children’s above-chance performance in the Binary with No Perceptual Interference condition, the Quaternary Relation with No Perceptual Interference condition, and the Quaternary Relation with Perceptual Interference condition demonstrated that 3- and 4-year-olds were able to find analogical matches when they were familiar with the relations involved and with the demands of perceptual interference and RI. The results were consistent with Richland et al. (2006). Second, the developmental pattern in the perceptual interference manipulation showed the effect of the introduction of perceptual interference. Although 3- and 4-year-olds can attend to and map analogical relations to some extent, children and even adults cannot fully resist the perceptual interference. Third, all age groups performed worse in Quaternary Relation conditions than in Binary Relation conditions. This provided evidence that the additional levels of RI caused greater difficulty in the analogical reasoning. The results also indicated that the methodological concern that children might have used a strategy of ignoring the broken table and broken mirror and solved the alignment based on the binary relation in the Quaternary Relation condition was not likely. This is because that would result in a lower level of RI and better performance in the Quaternary Relation condition than the Binary Relation condition.

Interestingly, a significant three-way interaction among age, perceptual interference, and RI indicated an age-related pattern in analogical reasoning. We found that different mechanisms may influence the interaction between RI and perceptual interference in children and adults. In children, the disruptive effect of interference is greatest when simple binary relations have to be mapped. Or, the disruptive effect of RI is greatest when the perceptual interference is absent. However, in adults, the disruptive effect of the perceptual interference increased when the more complex RI was present. Or, the disruptive effect of RI increased when perceptual interference was present. The different patterns of perceptual interference and RI between children and adults provided evidence for the claim that for children, the interactive effect of RI and perceptual interference was less than the sum of their respective singular effects. In contrast, for adults, the interactive effect of RI and interference exceeded the sum of their singular effects.

## Experiment 2

As mentioned above, the ability to identify perceptual interference develops earlier than the identification of semantic interference, which is acquired through learning. How do children develop from being affected by superficial perceptual interference to being affected by more abstract semantic interference? In Experiment 2, we used the same method as Experiment 1 but using semantic interference instead of perceptual interference to investigate the role of semantic interference and RI in the development of analogical reasoning. More importantly, we further investigated the comprehensive developmental mechanism between the two types of interference and RI in the development of analogical reasoning.

### Methods

#### Participants

Thirty 3- and 4-year-old children (15 females; *M* = 47 months, *SD* = 4.33 months), 27 5- and 6-year-old children (16 females; *M* = 66.3 months, *SD* = 5.88 months), and 40 adults (24 females; *M* = 21.0 years, *SD* = 1.33 years) took part in this experiment. All participants were recruited in Beijing, China. They were all right-handed and reported normal or corrected-to-normal vision without color blindness. None of the subjects had previously participated in similar experiments. Informed written consent from participants or parents was obtained. Children were given gifts and adults were paid for their participation.

#### Materials

**FIGURE 3:**
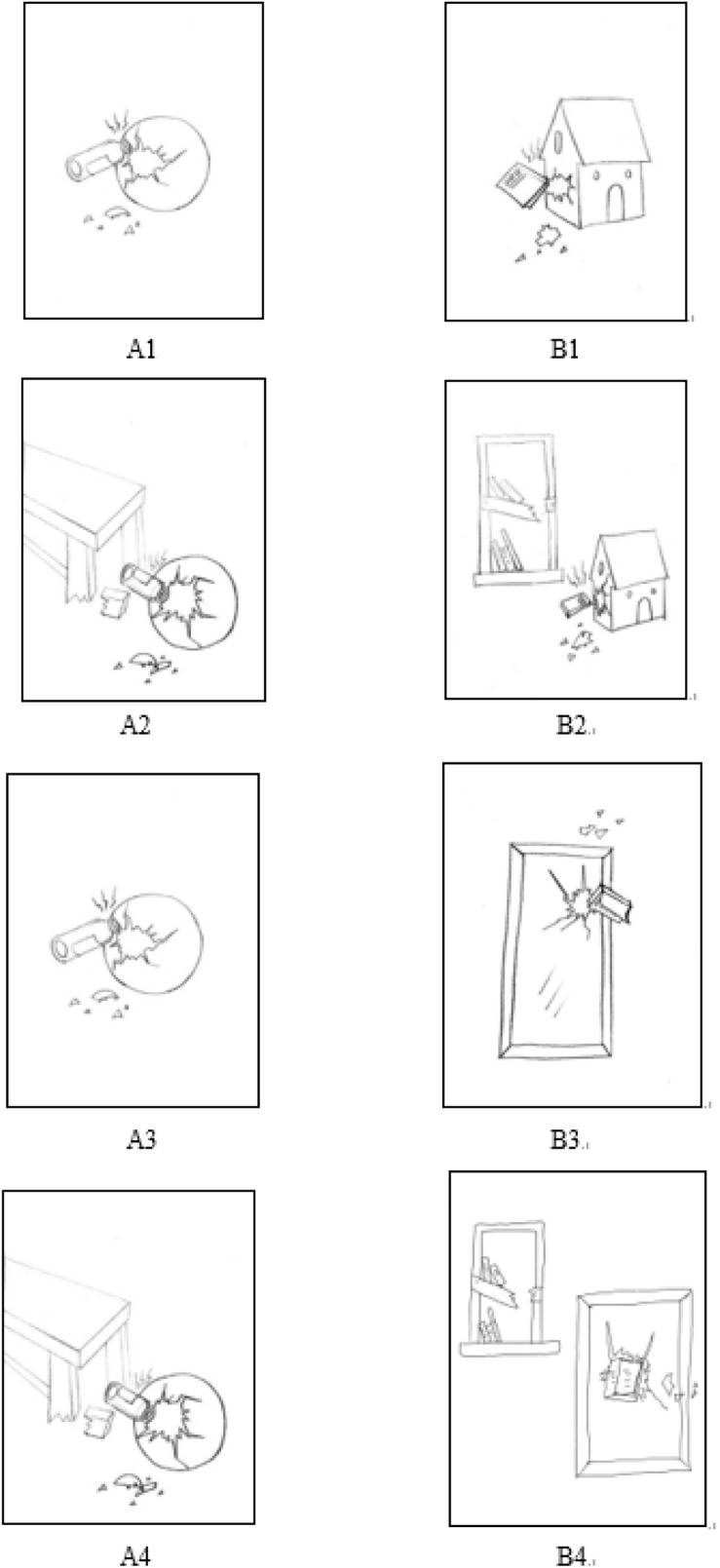
Examples of trials for the analogy scene in Experiment 2. Pictures labeled with “A” represent source pictures and pictures labeled with “B” represent target pictures. **(A1,B1)** shows a Binary Relation with No Semantic Interference analogy scene (the same as Experiment 1); **(A2,B2)** shows a Quaternary Relation with No Semantic Interference analogy scene (the same as Experiment 1); **(A3,B3)** shows a Binary Relation with Semantic Interference analogy scene [**(A3)** is the source scene: a glass bottle damaged a ball; **(B3)** is the target scene: a heavy book damaged a mirror]; **(A4,B4)** shows a Quaternary Relation with Semantic Interference analogy scene [**(A4)** is the source scene: the table leg was broken, which caused the glass bottle to damage the ball; **(B4)** is the target scene: the bookshelf was broken, which caused the heavy book to damage the mirror].

Figure 3 depicts an example of one scene from Experiment 2. There were 27 key relation groups including four conditions with 27 paired scenes in each condition. All paired scenes depicted everyday instances of common relations (such as damaging, chasing, pulling, etc.). These relations and objects were also familiar to preschool children. In half of the trials, each scene included a binary relation, and the remaining ones contained a quaternary relation (the RI factor). The binary relations and the quaternary relations were the same as in Experiment 1. In half of the trials, one object from a relation in the source scene was semantically similar to another object included in that relation in the target scene (the semantic interference factor). For example, the bottle (the object that causes damage) and the mirror (a damaged object) come from the same category of objects made from glass. In the other half of the trials, there was no semantic similarity between the source picture and the target picture.

Semantic interference emerged when proper mapping between analogically matching objects was incoherent with their categorical features. The semantic interference objects were inanimate objects (such as a mirror) or animate objects (such as animals). The spatial locations of the extra objects and semantic interference objects were controlled across paired scenes, as was the relative size of the objects. The semantic interference objects were varied to ensure that participants would not learn a pattern. For example, the correct answer was not always a human or not always in the same location or not always a bigger object.

To ensure that highlighted source objects were indeed semantically similar to the semantic interference objects, 12 undergraduates were asked to rate the semantic similarity between the highlighted source objects and other objects in the scene analogical tasks. For example, in the task in Figure 3, the instructions were as follows: Welcome to the picture evaluation! Please first look at all the pictures carefully. Then, please separately evaluate the semantic similarity (such as shared categorical features etc.) between the glass bottle and the following objects: the ball, the mirror, the book, the bookshelf, the table, the dollhouse on a Likert seven-point scale. In all scene groups, the highlighted objects were assessed as being semantically similar to the semantic interference objects with a score of 5.2 or more on the 1–7 scale. The highlighted objects were assessed as being semantically similar to other objects (except for highlighted objects) with a score of 2.8 or less on the scales. Furthermore, in order to clarify whether highlighted objects were perceptually similar to the semantic interference objects or other objects, the 12 undergraduates were asked to rate the perceptual similarity between the semantic interference objects and other objects in the scene analogical tasks. For example, in the task in Figure 3, the instructions were as follows: Please separately evaluate the perceptual similarity (such as similar shape, similar color, etc.) between the glass bottle and the following objects – the ball, the mirror, the book, the bookshelf, the table, the dollhouse on a Likert seven-point scale. The highlighted objects were perceptually similar to all objects (including the semantic interference objects) with a score of 2.8 or less on the scale. According to Thibaut et al. (2011), these indicated a valid manipulation of semantic interference.

#### Procedure

The procedure in Experiment 2 was identical to Experiment 1, except that semantic interference was included in the test materials instead of perceptual interference. None of the children’s data was excluded due to meeting the criterion of Richland et al. (2006).

### Results

The descriptive statistics for the participant’s accuracies are presented in Table 2. A 3 (Age Group: 3- and 4-year-olds, 5- and 6-year-olds, or adults) × 2 (RI: Binary Relations or Quaternary Relations) × 2 (Semantic Interference: No Semantic Interference or Semantic Interference) mixed ANOVA was conducted on the accuracies. The results revealed that all main effects were significant. The main effect of age group was significant, *F*(2,94) = 188.509, *p* < 0.001, η*^2^* = 0.800; 3- and 4-year-olds (*M* = 0.601 ± 0.009) and 5- and 6-year-olds (*M* = 0.610 ± 0.010) responded correctly less frequently than adults (*M* = 0.790 ± 0.007). The main effect of RI was significant, *F*(1,94) = 205.123, *p* < 0.001, η*^2^* = 0.686; participants’ accuracy decreased when RI increased from two (*M* = 0.713 ± 0.006) to four (*M* = 0.615 ± 0.006) dimensions. The main effect of semantic interference was also significant, *F*(1,94) = 251.713, *p* < 0.001, η*^2^* = 0.728; participants responded correctly less frequently when semantic interference was present (*M* = 0.610 ± 0.006) than when it was absent (*M* = 0.718 ± 0.006).

**TABLE 2:** Mean accuracies and standard deviations of the Scene Analogy Task for 3- and 4-year-olds, 5- and 6-year-olds, and adults in Experiment 2.

Age	Binary relations		Quaternary relations	
		
	No semantic interference	Semantic interference	No semantic interference	Semantic interference
3- and 4-year-olds (*n* = 30)	0.696 ± 0.069	0.660 ± 0.090	0.552 ± 0.092	0.497 ± 0.092
5- and 6-year-olds (*n* = 27)	0.741 ± 0.054	0.533 ± 0.059	0.622 ± 0.062	0.507 ± 0.079
Adults (*n* = 40)	0.869 ± 0.072	0.778 ± 0.056	0.830 ± 0.066	0.684 ± 0.076

The interaction between age group and semantic interference was significant, *F*(2,94) = 22.621, *p* < 0.001, η*^2^* = 0.325. A simple effect test in each semantic interference condition (using a Bonferroni adjustment) showed a significant main effect of age group in the no semantic interference condition, *F*(2,94) = 151.489, *p* < 0.001, η*^2^* = 0.763, as well as in the semantic interference condition, *F*(2,94) = 117.175, *p* < 0.001, η*^2^* = 0.714. Tukey’s HSD tests showed that in the no semantic interference condition, 3- and 4-year-olds performed worse than 5- and 6-year-olds and adults (*p* < 0.005); 5- and 6-year-olds performed worse than adults (*p* < 0.001). In the semantic interference condition, 5- and 6-year-olds performed worse than 3- and 4-year-olds and adults (*p* < 0.005); 3- and 4-year-olds performed worse than adults (*p* < 0.001). Moreover, the interaction between age group and RI was significant, *F*(2,94) = 16.944, *p* < 0.001, η*^2^* = 0.265. A simple effect test in each age group (using a Bonferroni adjustment) showed a significant main effect of RI in 3- and 4-year-olds, *F*(1,94) = 161.358, *p* < 0.001, η*^2^* = 0.632; in 5- and 6-year-olds, *F*(1,94) = 32.550, *p* < 0.001, η*^2^* = 0.257; and in adults, *F*(1,94) = 40.467, *p* < 0.001, η*^2^* = 0.301. Tukey’s HSD tests showed that in all age groups, participants performed significantly worse when RI increased from two to four dimensions (*p*’s < 0.001).

**FIGURE 4:**
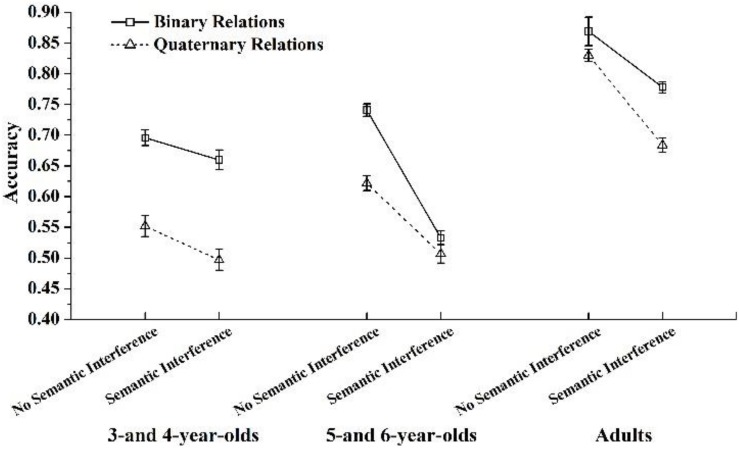
Accuracies as a function of relational integration and semantic interference across age groups.

We also found a significant three-way interaction among age group, RI, and semantic interference, *F*(2,94) = 11.831, *p* < 0.001, η*^2^* = 0.201. To further precisely characterize age-related differences in patterns of accuracy on the task, a test of RI × semantic interference was conducted on each age group separately (Figure 4).

Results showed that for 3- and 4-year-olds, there was a main effect of RI, *F*(1,29) = 148.366, *p* < 0.001, η*^2^* = 0.836; and a main effect of semantic interference, *F*(1,29) = 14.425, *p* < 0.001, η*^2^* = 0.332; whereas the interaction between RI and semantic interference was not significant.

For 5- and 6-year-olds, there was a main effect of RI, *F*(1,26) = 31.479, *p* < 0.001, η*^2^* = 0.548; a main effect of semantic interference, *F*(1,26) = 161.509, *p* < 0.001, η*^2^* = 0.861; and a significant interaction between RI and semantic interference, *F*(1,26) = 23.558, *p* < 0.005, η*^2^* = 0.475. A simple effect test in each semantic interference condition (using a Bonferroni adjustment) showed a significant main effect of RI in the no semantic interference condition, *F*(1,26) = 59.567, *p* < 0.001, η*^2^* = 0.696; and in the semantic interference condition, *F*(1,26) = 2.557, *p* > 0.05. Tukey’s HSD tests showed that in the no semantic interference condition, children performed significantly worse when RI increased from two to four dimensions (*p* < 0.001); in the semantic interference condition, there was no significant difference between the binary relations condition and the quaternary relations condition (*p* > 0.05).

For adults, there was a main effect of RI, *F*(1,39) = 44.362, *p* < 0.001, η*^2^* = 0.532; a main effect of semantic interference, *F*(1,39) = 123.685, *p* < 0.001, η*^2^* = 0.760; and a significant interaction between RI and semantic interference, *F*(1,39) = 6.153, *p* < 0.05, η*^2^* = 0.136. A simple effect test in each semantic interference condition (using a Bonferroni adjustment) showed a significant main effect of RI in the no semantic interference condition, *F*(1,39) = 8.475, *p* < 0.001, η*^2^* = 0.179; and in the semantic interference condition, *F*(1,39) = 33.250, *p* < 0.001, η*^2^* = 0.460. Tukey’s HSD tests showed that in the no semantic interference condition, adults performed significantly worse when RI increased from two to four dimensions (*p*’s < 0.05). In the semantic interference condition, adults performed significantly worse when RI increased from two to four dimensions (*p*’s < 0.001).

We also calculated the difference in accuracy between no semantic interference and semantic interference in the Binary Relation condition as well as the difference in accuracy between no semantic interference and semantic interference in the Quaternary Relation condition across the three age groups. Paired *t* tests on these two differences in accuracy were conducted across the three age groups. The results showed that for 3- and 4-year-olds, the difference in accuracy between no semantic interference and semantic interference in the Binary Relation condition was not significantly different from that in the Quaternary Relation condition, *t*(29) = −0.865, *p* > 0.05; for 5- and 6-year-olds, the difference in accuracy between no semantic interference and semantic interference in the Binary Relation condition was significantly larger than that in the Quaternary Relation condition, *t*(26) = 4.854, *p* < 0.001. For adults, the difference in accuracy between no semantic interference and semantic interference in the Binary Relation condition was significantly smaller than that in the Quaternary Relation condition, *t* (39) = −2.480, *p* = 0.018.

These three patterns revealed changes with age. All age groups showed strong effects of semantic interference and RI. Interestingly, the interactive effect of semantic interference and RI was significant for 5- and 6-year-olds and adults, rather than 3- and 4-year-olds. Specifically, for 5- and 6-year-olds, the difference between semantic interference and no semantic interference was larger when RI was simple (i.e., binary relations) rather than complex (i.e., quaternary relations). Similarly, the difference in the accuracy between the Binary Relations and Quaternary Relations conditions was larger when semantic interference was absent than when it was present. However, for adults, the difference in accuracy between semantic interference and no semantic interference was larger when RI was complex (i.e., quaternary relations) rather than simple (i.e., binary relations). Similarly, the difference between accuracy in the Binary Relation and Quaternary Relation conditions was larger when semantic interference was present than when it was absent.

The chance level differed across conditions (Richland et al., 2006). In the current study, it ranged from 50% (Binary Relation with No Semantic Interference and Binary Relation with Semantic Interference) to 33% (Quaternary Relation with No Semantic Interference and Quaternary Relation with Semantic Interference). A paired *t* test demonstrated that 3- and 4-year-olds were above the level of chance for the Binary Relation with No Semantic Interference, *t* (29) = 15.658, *p* < 0.001; Binary Relation with Semantic Interference, *t* (29) = 9.715, *p* < 0.001; Quaternary Relation with No Semantic Interference, *t* (29) = 13.64, *p* < 0.001; and Quaternary Relation with Semantic Interference conditions, *t* (29) = 9.722, *p* < 0.001. This revealed that 3- and 4-year-olds understand the analogical relationships and can reason according to relational similarity; 5- and 6-year-olds and adults were above the level of chance for all conditions.

#### Combined Analyses

To compare the effects of perceptual interference and semantic interference on the development of analogical reasoning, we calculated the differences in accuracy by subtracting the accuracy scores in the without interference condition from the accuracy score in the with interference condition. The differences in accuracy represented the degree of interference.

**FIGURE 5:**
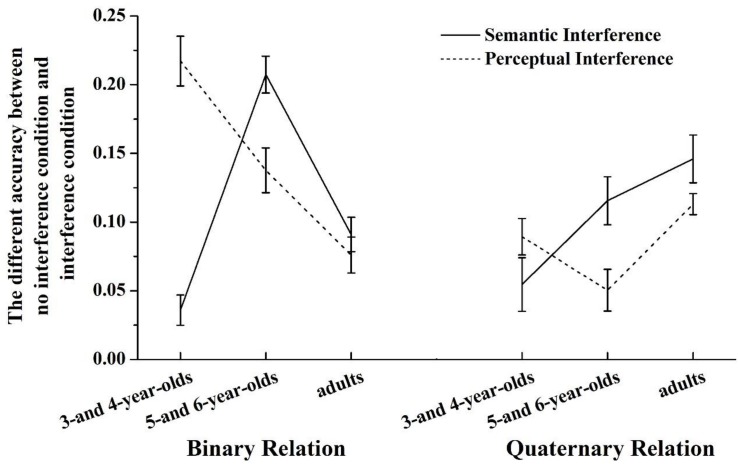
The difference in accuracy between the no interference condition and interference condition for Experiment 1 and Experiment 2 across relational integration, age, and interference type.

A 2 (RI: Binary Relation or Quaternary Relation) × 2 (Interference Type: perceptual interference or semantic interference) × 3 (Age: 3- and 4-year-olds, 5- and 6-year-olds, adults) mixed ANOVA was conducted on differences in accuracy in the without interference condition subtracting the interference condition (Figure 5). RI was the within-subject variable, while Interference Type and Age were the between-subject variables. Results showed that the main effect of RI was significant, *F*(1,188) = 14.213, *p* < 0.001, η*^2^* = 0.070. The effect of interference decreased when RI increased from two to four dimensions. The main effect of age was also significant, *F*(2,188) = 3.394, *p* < 0.05, η*^2^* = 0.035; 5- and 6-year-olds showed a smaller difference between interference and no interference than 3- and 4-year-olds and adults (*p*’s < 0.05). There was no significant difference between 3- and 4-year-olds and adults (*p* > 0.05). We found a significant three-way interaction among age, RI, and interference type, *F*(2,188) = 7.034, *p* < 0.001, η*^2^* = 0.07.

We further conducted a test of the effect of Interference Type with Age on each type of RI (using a Bonferroni adjustment). Results showed that for the Binary Relations condition, there was a main effect of interference type, *F*(1,188) = 7.387, *p* < 0.01, η*^2^* = 0.038; a main effect of age, *F*(2,188) = 19.422, *p* < 0.001, η*^2^* = 0.171; and a significant interaction between age and interference type, *F*(2,188) = 39.023, *p* < 0.001, η*^2^* = 0.293, A test of the simple effect of interference type on each age group (using a Bonferroni adjustment) showed a significant effect of interference type on 3- and 4-year-olds, *F*(1,188) = 74.311, *p* < 0.001, η*^2^* = 0.283; and on 5- and 6-year-olds, *F*(1,188) = 9.861, *p* < 0.005, η*^2^* = 0.05. Tukey’s HSD tests showed that 3- and 4-year-olds showed a larger difference between interference and no interference when perceptual interference was present rather than when semantic interference was present (*p* < 0.001). However, 5- and 6-year-olds showed a larger difference between interference and no interference when semantic interference was present rather than when perceptual interference was present (*p* < 0.001). For adults, there was no significant difference between interference and no interference whether perceptual interference was present or semantic interference was present.

For the Quaternary Relations condition, there was a main effect of age, *F*(2,188) = 8.445, *p* < 0.001, η*^2^* = 0.082; and a significant interaction between age and interference type, *F*(2,188) = 4.843, *p* < 0.01, η*^2^* = 0.049. A test of the simple effect of interference type on each age group (using a Bonferroni adjustment) showed a significant effect of interference type on 5- and 6-year-olds, *F*(1,188) = 7.338, *p* < 0.005, η*^2^* = 0.038. Tukey’s HSD tests showed that 5- and 6-year-olds showed a larger difference between interference and no interference when semantic interference was present rather than when perceptual interference was present (*p* < 0.005). For 3- and 4-year-olds and adults, there was no significant difference between interference and no interference whether perceptual interference was present or semantic interference was present.

We also further conducted a test of the effect of Interference Type with RI on each age group. Results showed that for 3- and 4-year-olds, there was a main effect of RI, *F*(1,58) = 14.000, *p* < 0.001, η*^2^* = 0.194; a main effect of interference type, *F*(1,58) = 37.948, *p* < 0.001, η*^2^* = 0.396; and a significant interaction between RI and interference type, *F*(1,58) = 25.194, *p* < 0.001, η*^2^* = 0.303. A simple effect test in each RI condition (using a Bonferroni adjustment) showed a significant main effect of interference type in the Binary Relations condition, *F*(1,58) = 70.373, *p* < 0.001, η*^2^* = 0.548; and in the Quaternary Relations condition, *F*(1,58) = 2.092, *p* > 0.05. Tukey’s HSD tests showed that in the Binary Relations condition, 3- and 4-year-olds showed a larger difference between interference and no interference when perceptual interference was present rather than when semantic interference was present (*p* < 0.001). In the Quaternary Relations condition, there was no significant difference between interference and no interference conditions whether perceptual interference was present or semantic interference was present (*p* > 0.05).

For 5- and 6-year-olds, there was a main effect of RI, *F*(1,52) = 27.472, *p* < 0.001, η*^2^* = 0.346; a main effect of interference type, *F*(1,52) = 21.041, *p* < 0.001, η*^2^* = 0.288; and the interaction between RI and interference type was not significant, *F*(1,52) = 0.017, *p* > 0.05.

For adults, there was a main effect of RI, *F*(1,78) = 11.829, *p* < 0.005, η*^2^* = 0.132; the main effect of interference type was not significant, *F*(1,78) = 3.332, *p* > 0.05; and the interaction between RI and interference type was not significant, *F*(1,78) = 0.453, *p* > 0.05.

Taken together, for 3- and 4-year-olds, the degree of perceptual interference was larger than the degree of semantic interference in the Binary Relation condition, whereas there was no significant difference between the degree of two types of interference in the Quaternary Relation condition. Moreover, for 5- and 6-year-olds, the degree of semantic interference was larger than the degree of perceptual interference in both relation conditions. For adults, there was no difference between the degree of two types of interference in both relation conditions.

### Discussion

Experiment 2 investigated the role of semantic interference and RI in the development of analogical reasoning. The results of Experiment 2 were consistent with those of Experiment 1, which also revealed the main effects of age and RI. Unlike Experiment 1, the results in Experiment 2 demonstrated that declarative semantic-level interference can increase the difficulty in performing analogical reasoning tasks. This is a similar finding to that of Chuderska and Chuderski (2014).

We also found a significant three-way interaction among age, semantic interference, and RI, which indicated an age-related pattern in analogical reasoning. In contrast to Experiment 1, we only found a significant interaction between RI and semantic distractors in 5- and 6-year-olds, but not in 3- and 4-year-old children. This may be due to the differential time courses of development of perceptual knowledge and semantic knowledge in children.

Similar to Experiment 1, the interactive patterns of RI and interference in 5- and 6-year-olds and adults were different. Specifically, in the no semantic interference condition, 5- and 6-year-olds performed significantly worse when RI increased from two to four dimensions; there was no significant difference between the binary relations condition and the quaternary condition in the semantic interference condition. This suggested that for children, the interference resolution and relation integration depended on shared cognitive resources. If one factor required more cognitive demand (i.e., the RI became more complex or interference was present), limited resources were available for another factor (such as interference resolution or RI). However, adults performed significantly worse when RI increased from two to four dimensions whether or not there was semantic interference. This showed that for adults, the interference resolution and RI can cause the cumulative difficulty in analogical reasoning.

The combined analyses demonstrated that in contrast to the perceptual interference results in Experiment 1, the degree of semantic interference became larger at 5- and 6-year-olds not at 3- and 4-year-olds. These results suggest two possibilities. One is that 5- and 6-year-olds have already been influenced by more abstract and difficult semantic interference whether in a simple relation condition or a complex relation condition. Another possibility is that due to limited categorical knowledge, 3- and 4-year-olds cannot understand the abstract semantic connection between “glass bottle” and “mirror” when they complete the semantic interference scene task.

## General Discussion

The current study introduced RI and two types of interference (perceptual interference in Experiment 1, semantic interference in Experiment 2) into a scene analogical reasoning task for 3- and 4-year-olds, 5- and 6-year-olds, and adults. Significant main effects of perceptual interference, semantic interference, and RI were founded. These showed that the manipulation of perceptual interference, semantic interference, and RI was valid.

Apart from the expected main effects, we also found different patterns between perceptual interference and semantic interference in the development of analogical reasoning. Only considering the simple relation condition, in which children have already attended to and map basic analogical reasoning abilities, 3- and 4-year-olds encountered more difficulties in coping with perceptual interference rather than semantic interference. Moreover, for 5-and 6-year-olds, the degree of semantic interference was larger than the degree of perceptual interference. Previous studies have found that with increasing age, abstract-level semantic information rather than visually similar perceptual information played a more important role in cognitive development (Koutstaal, 2003; Burnside et al., 2017). The current results provided consistent evidence that 3- and 4-year-olds were unable to contextualize the semantic interference, but perceptual interference played a large role. The results showed that 5- and 6-year-olds, unlike 3- and 4-year-olds, can contextualize abstract semantic information.

For the first time, we found different interactions between interference and RI across different age groups. We found that for children, the disruptive effect of interference was larger in the Binary Relation condition than in the Quaternary Relation condition. In other words, the disruptive effect of RI was larger in the no interference condition than in the interference condition. There may be several possible reasons for the results. One possible explanation is working memory. Mean working memory capacity is usually estimated to be between three and four chunks (Cowan, 2001). In the Binary Relation condition, all objects in the analogical reasoning scene can be maintained in working memory, and the RI did not exceed the working memory capacity of the children. Thus, the interference is easily available for the mapping process. However, in the Quaternary Relationship condition, the requirement to actively maintain four elements in working memory and integrate the complicated relations simultaneously might have exceeded the working memory capacity of the children, as some children may have only been able to maintain two or three chunks. The effect of interference might not be as large as in the simple relation complexity condition. Another possibility is that the quaternary condition or interference condition made the respective aspects of the scenes more salient. For example, the larger effect of interference in the binary condition or the larger effect of the quaternary condition in the no interference condition may be attentional.

Furthermore, 3- and 4-year-olds only showed a significant interaction between RI and perceptual interference, not semantic interference. The results showed that 5- and 6-year-olds were affected by both perceptual and semantic interference with RI. These results might be due to the different time courses of development of perceptual knowledge and semantic knowledge. In adults, we found that the effect of the interference increased when the more complex RI was present, or, the difficulty effect of the RI increased when interference was present. These results are consistent with those of previous studies on adults (Cho et al., 2007). This may be due to adults’ well-developed working memory capacity. All objects of an analogical scene can be maintained, even in the Quaternary Relation condition. Thus, for adults, the interactive effect of RI and interference exceeded the sum of their respective singular effects.

A comparison of the interaction between interference and RI in children and adults suggests that for children, RI and interference resolution seem to share a common resource in analogical reasoning (Cho et al., 2007). If one disruptive factor needs more cognitive resources, there are limited resources available for another disruptive factor. However, for adults, RI and interference resolution seem to be independent facets of analogical reasoning. Therefore, interference and RI can decrease performance cumulatively.

### Limitations and Future Work

Although we argue that the performance in the Quaternary Relation condition was worse than that of the Binary Relation condition in both experiments and the results revealed the validity of RI manipulation to some extent, we cannot completely rule out the effect of visual noise. The number of objects in the Binary Relation condition differed from that of the Quaternary Relation condition. In the Binary Relation condition, it is not possible to tease apart how many relationships need to be considered from the influence of more visual objects to make sense of. Future work should control the number of objects in all conditions, varying relational complexity but not visual complexity. For instance, an object involved in the relation could be replaced with one that was not involved.

Additional studies are also needed to investigate what kind of errors children make. This is important as these other kinds of errors can be informative when participants do not make the expected perceptual/semantic errors. Such studies could also reveal the developmental differences in different kinds of errors. Furthermore, further studies are needed to elucidate the mechanisms by which RI and other contributors interact with factors involved in development, such as goal-driven selection (Starr et al., 2018), relational comparison (Begolli et al., 2018), and relational language (Gentner et al., 2011; Son et al., 2012; Christie and Gentner, 2014; Fyfe et al., 2015; Gentner, 2016).

In summary, the findings of the current study make a theoretical contribution and have practical implications. From the theoretical perspective, this study attempted to explore and compare the influence of perceptual interference, semantic interference, and RI on the analogical reasoning of children and adults. Moreover, our study indicated that cognitive resources in children’s analogical reasoning are shared between RI and interference resolution. Furthermore, from the applied perspective, researchers should consider strategies for alleviating RI and inhibiting the impact of irrelevant stimuli on children, such as representing the source and target simultaneously.

## Data Availability Statement

The datasets generated for this study are available on request to the corresponding author.

## Ethics Statement

This study was carried out in accordance with the recommendations of the School of Psychology Ethics Committee at Beijing Normal University with written informed consent from subjects or subjects’ parents. All experimental procedures were approved by the School of Psychology Ethics Committee at Beijing Normal University and conformed to the Declaration of Helsinki.

## Author Contributions

XY contributed to conception and design, on acquisition and interpretation of data, and on drafting of the manuscript. YC contributed to conception and design, and on interpretation of the data. LG contributed to drafting of the manuscript. XZ contributed on interpretation of the data. CH contributed to making the experimental materials and entering the data.

## Conflict of Interest

The authors declare that the research was conducted in the absence of any commercial or financial relationships that could be construed as a potential conflict of interest.
